# Fumarate and nitrate reduction regulator (FNR) modulates hypermucoviscosity and virulence in hypervirulent *Klebsiella pneumoniae* through anaerobic adaptation

**DOI:** 10.1080/21505594.2025.2536186

**Published:** 2025-07-28

**Authors:** Yidan Xu, Ruolan Guo, Ruomei Wang, Zhe Su, Yulu Wu, Chengbin Xie, Pinjia Wang

**Affiliations:** a School of Laboratory Medicine, Chengdu Medical College, Chengdu, Sichuan, China; b Department of Laboratory Medicine, Sichuan Provincial Maternity and Child Health Care Hospital, Chengdu, Sichuan, China; c Department of Laboratory Medicine, The Affiliated Women’s and Children’s Hospital of Chengdu Medical College, Chengdu, Sichuan, China

**Keywords:** Hypervirulent *Klebsiella pneumoniae*, fumarate and nitrate reduction regulator, FNR, hypermucoviscosity, anaerobic adaptation, virulence

## Abstract

Hypervirulent *Klebsiella pneumoniae* (hvKP), a pathogen responsible for severe invasive infections, exhibits a hypermucoviscosity (HMV) phenotype that is closely associated with its virulence. While fumarate and nitrate reduction regulator (FNR), a global transcription regulator, is critical for bacterial adaptation to hypoxic conditions, its role in hvKP pathogenicity remains unexplored. This study demonstrates that FNR modulates the HMV phenotype and virulence of the hvKP strain NTUH-K2044 under anaerobic conditions. Through targeted deletion and complementation of the *fnr* gene, combined with phenotypic, molecular, cellular, and animal infection assays, we show that FNR positively regulates the HMV phenotype. Notably, this regulation is independent of several genes previously implicated in HMV formation, including *rmpA*, *rmpA2*, *wzy-K1* (*magA*), *rmpC*, and *rmpD*. In the absence of *fnr*, the HMV phenotype was abolished, while the transcript levels of these genes increased significantly, suggesting a compensatory or indirect regulatory mechanism that warrants further investigation. Functionally, FNR-mediated HMV enhanced bacterial resistance to phagocytosis and serum killing while suppressing host colonization features such as fimbriae formation, biofilm production, and epithelial cell adhesion. In animal infection models, FNR also contributed positively to hvKP virulence. These findings highlight the role of FNR in regulating the HMV phenotype and virulence in hvKP, facilitating host adaptation and immune evasion. Targeting FNR may thus represent a promising strategy for the development of novel therapeutics.

## Introduction

*Klebsiella pneumoniae*, a clinically significant gram-negative bacillus, functions as both an opportunistic and nosocomial pathogen. Based on virulence and pathogenicity, *K. pneumoniae* strains are classified as classical (cKP) or hypervirulent (hvKP). The cKP strains are primarily implicated in hospital-acquired infections in immunocompromised patients, such as pneumonia and bacteraemia, and they are often characterized by multidrug resistance but relatively low virulence [1,2]. In contrast, hvKP strains exhibit heightened virulence, causing severe community-acquired invasive diseases, including pyogenic liver abscesses and meningitis, despite generally retaining antibiotic susceptibility [1]. The global incidence of hvKP infections is rising, with prevalence in China reported between 8.3% and 73.9%, reflecting a considerable regional disease burden [3,4]. Notably, hvKP can cause rapid progression to life-threatening metastatic complications through multiple virulence factors in otherwise healthy hosts [2], marked by poor prognosis and high mortality, thereby posing significant therapeutic challenges.

A hallmark of hvKP is the hypermucoviscosity (HMV) phenotype, characterized by excessive mucoid production that allows the formation of a viscous string exceeding 5 mm when stretched from a bacterial colony using an inoculation loop [5]. This phenotype reflects a surface coating enriched in exopolysaccharides with higher viscosity than typical capsular material [1]. Early studies linked HMV to capsule polysaccharide (CPS) overproduction, regulated by *rmpA*, *rmpA2*, and *wzy-K1* (*magA*) genes [6–9]. However, recent evidence suggests that HMV is not solely dependent on capsule synthesis [10,11]. Additional regulatory genes, namely, *rmpC* and *rmpD*, located near the *rmpA* locus, have distinct yet overlapping roles in modulating HMV and capsule production. Deletion of *rmpA* abolished both traits, indicating its essential regulatory function. In contrast, the Δ*rmpC* mutant retained HMV but had reduced capsule production, while the Δ*rmpD* mutant lost HMV but preserved capsule synthesis, identifying *rmpC* and *rmpD* as specific regulators of capsule production and HMV, respectively. These findings support the view that HMV and capsule expression are independently regulated traits. The HMV phenotype is closely associated with hypervirulence, and it enhances the resistance of hvKP strains to neutrophil- and macrophage-mediated phagocytosis as well as complement-mediated serum killing [1,12,13]. Moreover, recent studies suggest that the antiphagocytic effect is primarily attributable to HMV rather than capsule overproduction [14], establishing HMV as a key virulence determinant.

*K. pneumoniae* is a facultative anaerobe commonly residing in the gastrointestinal tract, where its ability to sense and respond to oxygen fluctuations is essential for survival. This adaptation is orchestrated by complex regulatory networks, including the fumarate and nitrate reduction regulator (FNR), an oxygen-sensitive transcriptional regulator that contains iron-sulfur (Fe-S) clusters. Under anaerobic conditions, FNR dimerizes via [4Fe-4S]^2+^ clusters and binds to the “TTGATnnnnnnATCAA” promoter motif to activate genes involved in anaerobic metabolism [15]. In the presence of oxygen, the Fe-S cluster transitions to [2Fe-2S]^2+^, rendering FNR inactive [16]. In addition to metabolic regulation, FNR influences bacterial motility [17], toxin production [18], and antibiotic susceptibility [19]. These roles vary across species, underscoring the need to elucidate FNR function specifically in *K. pneumoniae*.

Given the important role of FNR in regulating metabolism, motility, and virulence in diverse bacteria, we aimed to determine whether FNR influences the hypervirulence of hvKP strains. Our results show that FNR positively regulates HMV development in the hvKP strain NTUH-K2044 (K1) under anaerobic conditions. Importantly, this regulation occurs independently of the transcriptional control of *rmpA*, *rmpA2*, *magA*, *rmpC*, and *rmpD*. The increased mucus production conferred by FNR enhances resistance to phagocytosis and serum killing while reducing fimbriae expression, biofilm formation, and epithelial adhesion. Animal infection models further confirm the role of FNR in enhancing hvKP virulence, underscoring its critical contribution to pathogenicity. These findings suggest that therapeutic strategies targeting FNR-regulated pathways may hold promise for managing hvKP-associated infections.

## Materials and methods

### Strains, plasmids, primers, media, and culture conditions

K. *pneumoniae* NTUH-K2044, a hypermucoviscous strain of capsular serotype K1 commonly used as a model for studying hvKP, was obtained from Hangzhou Baosai Biotechnology Co., Zhejiang, China. *Escherichia coli* strains S17–1 λpir and DH5α were used for cloning. *Saccharomyces cerevisiae* ATCC 9763 was employed in the yeast cell agglutination assay. The plasmids and primers utilized in this study are detailed in Table S1 and Table S2 of the supplementary materials. When required for SacB counterselection, 10% sucrose was added to Luria-Bertani (LB) agar plates. For the selection of transformants and conjugants, chloramphenicol (Cm) and ampicillin (Amp) were used at final concentrations of 34 µg/mL and 30 µg/mL, respectively. Aerobic cultures were grown in LB broth at 37°C with shaking at 200 rpm until the logarithmic phase was reached. For anaerobic growth, bacteria were inoculated into LB broth and incubated statically at 37°C to the logarithmic phase in an anaerobic chamber containing a gas mixture of 5% CO_2_, 10% H_2_, and 85% N_2_.

### Construction of fnr mutant strains

A mutant strain named Δ*fnr* was generated by deleting 753 bp of the *fnr* coding region from the WT NTUH-K2044 strain via a homologous recombination strategy mediated by a suicide plasmid vector. Briefly, a fusion fragment of the upstream and downstream homology arms of *fnr* was seamlessly cloned and inserted into pH73, a suicide plasmid vector containing a counter selectable *sacB* marker and the Cm^R^ gene. The recombinant plasmid was transformed into *E. coli* S17–1 λ*pir* competent cells, which were subsequently screened to obtain positive clones, and the recombinant plasmid in the positive clones was subsequently transferred into NTUH K2044 via conjugation. Only if the recombinant plasmid was successfully integrated into the NTUH K2044 chromosome could the transconjugant carry both the Cm^R^ gene and the Amp^R^ gene. Colonies grown on an LB plate containing Amp (30 µg/mL) and Cm (34 µg/mL) were inoculated onto an LB agar plate containing 10% sucrose, and then, colonies grown on a sucrose plate were transferred to LB broth for expanded culture before spot inoculation onto an LB plate containing Cm (34 µg/mL). Colonies growing on sucrose agar plates but sensitive to Cm were screened, and the deletion of *fnr* was subsequently verified via PCR and whole-genome sequencing.

### Construction of fnr-complemented strains

Construction of *fnr*-complemented strains was carried out by first amplifying a DNA fragment containing the *fnr* promoter and coding sequence using the primer pair FNR-B95-F/FNR-B95-R. The pB95 plasmid was used as a template and linearized via PCR with the primer pair B95-FNR-F/B95-FNR-R. In seamless cloning, the promoter-containing *fnr* amplification product was ligated into the reverse-amplified pB95 product, which was then transformed into *E. coli* DH5α competent cells and plated on LB agar containing Cm (34 µg/mL). Positive clones were identified using the primers pB95-JD-F/M13-JD-R. The resulting recombinant plasmid was subsequently electroporated into Δ*fnr*, and the complemented mutant strain was designated C-Δ*fnr*.

### String test and mucoviscosity assay

The string test was conducted by gently stretching an anaerobically cultured colony grown on blood agar with an inoculation loop. A viscous string exceeding 5 mm was considered a positive result. For the mucoviscosity assay, 1 mL of bacterial culture, adjusted to an optical density (OD_600_) of 1.0, was centrifuged at 1000 × g for 5 minutes. The supernatant was carefully aspirated, and its OD_600_ was measured. HMV strains exhibit reduced pellet compaction after centrifugation owing to their high mucilage content, resulting in an elevated absorbance of the supernatant.

### Phagocytosis assay

The phagocytosis of *K. pneumoniae* by macrophages was assessed using a modified version of a previously described method [20]. Murine RAW264.7 cells were cultured in DMEM (Pricella Biotechnology, Wuhan, China) supplemented with 10% heat-inactivated fetal bovine serum (FBS) at 37°C in a 5% CO_2_ incubator. Cells (1 × 10^5^/well) were seeded in 12-well plates and cocultured with anaerobically grown *K. pneumoniae* strains at a multiplicity of infection (MOI) of 100 for 4 hours under anaerobic conditions. After incubation, wells were washed three times with PBS (pH 7.4), followed by the addition of 1 mL of DMEM containing gentamicin (100 µg/mL) to eliminate extracellular bacteria. After 1 hour, cells were washed and lysed with 200 µL of 0.5% Triton X-100 for 5 minutes. An additional 800 µL of PBS was added, and the lysate was homogenized, serially diluted, and plated on LB agar. The plates were then incubated overnight at 37°C to facilitate the quantification of colony-forming units (CFUs) per unit volume. The phagocytosis rate was expressed as the ratio of the number of bacteria in the cell to the initial number of bacteria multiplied by 100%. Each assay was performed in triplicate with three biological replicates.

### Serum killing assay

The serum killing assay was carried out according to previous methods with slight modifications [21]. Bacteria were inoculated in LB broth and cultured under anaerobic conditions to log phase. After three washes with PBS, the concentration of the bacterial suspension was adjusted to 1 × 10^6^ CFU/mL with PBS. The bacterial suspension (250 μL) was combined with healthy human serum (750 μL) and incubated under anaerobic conditions for 15 minutes. The mixture was subsequently diluted to a certain concentration and spread onto LB agar plates. Colonies were counted after incubation. Survival was expressed as the number of live bacteria after incubation with human serum relative to the initial bacterial count, multiplied by 100%. The experiment was repeated three times.

### Quantitative analysis of uronic acid

Uronic acid, a major component of the extracellular polysaccharides produced by *K. pneumoniae*, was quantified using the concentrated sulfuric acid – m-hydroxylamine method [20,22]. Bacterial strains were grown anaerobically to the logarithmic phase, harvested by centrifugation, and resuspended in PBS to a 1 McFarland standard. Polysaccharides were extracted from 10 mL of suspension using a bacterial polysaccharide extraction kit (Solarbio, Beijing, China). For quantification, D-glucuronic acid standards were prepared. Extracted samples were treated with precooled ammonium sulfamate and concentrated sulfuric acid containing sodium tetraborate. Absorbance was measured at 525 nm, and uronic acid concentration was determined using a standard curve. All of the assays were conducted in triplicate.

### Yeast cell agglutination assay

The yeast agglutination assay was used to assess type 1 fimbriae expression in *K. pneumoniae* strains, as previously described [23]. Strains were cultured in LB broth under anaerobic conditions until the OD_600_ reached 0.6. Bacterial cultures were then mixed with a 5% (w/v) suspension of *Saccharomyces cerevisiae* cells prepared in PBS. The mixture was applied to a slide, and agglutination was assessed after 10 minutes. All of the assays were performed in triplicate.

### Biofilm formation assay

Biofilm formation was evaluated using crystal violet staining [24]. Anaerobically grown bacterial cultures were adjusted to 0.5 McFarland using PBS. Then, 10 µL of bacterial suspension and 190 µL of LB broth were added to each well of a 96-well plate. Negative control wells contained 200 µL of LB broth alone. Plates were incubated anaerobically at 37°C for 24 hours. Following incubation, the medium was removed, and biofilms were fixed with 200 µL of methanol for 15 minutes. Wells were stained with 200 µL of 1% crystal violet for 20 minutes, rinsed with saline, and decolorized with 160 µL of anhydrous ethanol for 10 minutes. Absorbance was measured at 570 nm using a microplate reader. Each assay was performed in triplicate with three biological replicates.

### Cell adhesion assay

Adhesion of *K. pneumoniae* to epithelial cells was assessed with modifications from a previously reported method [20]. A549 human alveolar basal epithelial cells were cultured in Ham’s F-12K medium (Pricella Biotechnology, Wuhan, China) supplemented with 10% FBS at 37°C. Cells were seeded at 8 × 10^4^ cells per well in 24-well plates and incubated for 24 hours. Bacterial strains were added at an MOI of 100 and cocultured with A549 cells for 4 hours under anaerobic conditions. Wells were then washed three times with PBS to remove nonadherent bacteria. Cells were lysed with 1 mL of 0.1% Triton X-100 for 5 minutes. The lysate was subjected to serial dilution and spread onto LB agar plates. CFUs were counted after overnight incubation at 37°C. All of the assays were performed in triplicate.

### Quantitative reverse transcription PCR (qRT – PCR)

Total RNA was extracted from anaerobically cultured bacteria in the logarithmic phase using the SteadyPure Universal RNA Extraction Kit (Accurate Biology, Changsha, China). Genomic DNA was removed using the Evo M-MLV Reverse Transcription Premix Kit (Accurate Biology), and cDNA was synthesized according to the manufacturer’s instructions. qRT – PCR was performed using the SYBR Green Pro Taq HS premixed qRT – PCR Kit (Accurate Biology) on a CFX96 real-time PCR system (Bio-Rad, Hercules, CA, USA). Relative gene expression was analyzed using the 2^–ΔΔCt^ method, with 16S rRNA as the endogenous control. All of the reactions were performed in triplicate.

### Survival of *Galleria mellonella*

*Galleria mellonella* larvae (Huiyu De Biological Co., Ltd., Tianjin, China), weighing 250–350 mg, were used to assess virulence. Larvae were injected with 10 µL of *K. pneumoniae* suspension at 10^8^ CFU/mL (high dose) or 10^5^ CFU/mL (low dose) via the left proleg, resulting in inocula of 10^6^ and 10^3^ CFU, respectively. Control groups included untreated larvae, needle puncture only, and PBS-injected controls. Sample size was determined using G*Power 3.1 (effect size = 1.0, α = 0.05, power = 80%), requiring at least 8 larvae per group; 10 were used to account for attrition. Only larvae with intact cuticles and normal pigmentation were included. Randomization was used for group allocation. Larvae were incubated in the dark at 37°C, and mortality was recorded every 12 hours over 120 hours. Survival curves were constructed accordingly.

### In vivo blood invasion and phagocytosis assay

Bloodstream invasion and phagocytic response were assessed in 4–5-week-old female BALB/c mice (Dashuo Biotechnology Co., Ltd., China), maintained in a specific pathogen-free (SPF) facility (22 ± 2°C; 50 ± 10% humidity). Group size was determined by G*Power 3.1 (effect size = 1.2, α = 0.05, power = 80%), with 12 mice per group. Mice were block-randomized into three groups: WT, Δ*fnr*, and control. Mice were injected intraperitoneally with 100 µL of bacterial suspension (10^3^ CFU) or saline (control). At 12 and 36 hours post-infection, five mice per group were randomly selected and euthanized under isoflurane anaesthesia via cervical dislocation. Blood samples (100 µL) were plated on LB agar, incubated for 18 hours at 37°C, and CFUs were enumerated. For phagocytosis analysis, 3 mL of sterile saline was injected into the left peritoneal cavity of mice in each group, followed by gentle massage. Lavage fluid was collected from the right side, Gram-stained, and examined microscopically for phagocytes and bacterial presence. All of the procedures were performed under aseptic conditions.

### Animal ethics statement

All of the animal experiments were approved by the Animal Welfare Ethics Committee of Chengdu Medical College (Approval No. [2024] No. 046) and conducted in compliance with the Guide for the Care and Use of Laboratory Animals (Ministry of Science and Technology, P.R. China) and the ARRIVE guidelines.

### Statistical analysis

Statistical analysis was performed using Prism 9.3.1 (GraphPad Software, La Jolla, CA, USA). Data are presented as mean ± standard deviation (SD). Comparisons between groups were evaluated using unpaired *t*-tests or one-way ANOVA, as appropriate. Survival data were analyzed using the log-rank (Mantel – Cox) test. A *p*-value < 0.05 was considered to be statistically significant.

## Results

### Deletion of fnr inhibits the HMV phenotype in hvKP

**Figure 1.**
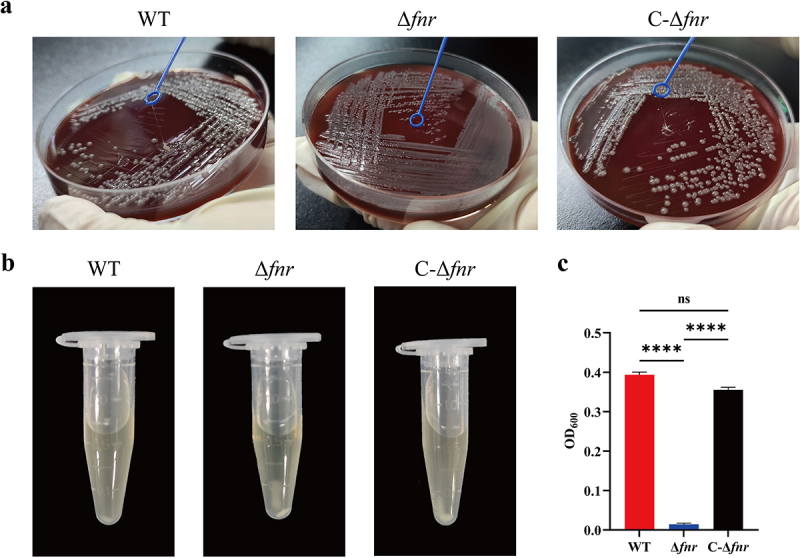
FNR positively regulates the HMV phenotype in the hvKP strain. (a) In the string test, the WT and C-Δ*fnr* strains produced viscous strings exceeding 5 mm from the colony. (b, c) In the sedimentation assay, the WT and C-Δ*fnr* strains remained suspended after centrifugation, whereas the Δ*fnr* mutant settled at the bottom of the tube, and its supernatant OD_600_ value was significantly lower than that of WT and C-Δ*fnr*. ****p < 0.0001, ns: not significant.

The *fnr* coding sequence was deleted from the wild-type (WT) strain via suicide vector-mediated homologous recombination, resulting in the Δ*fnr* mutant (Appendix S1, Figure S1a – e). Plasmids containing the *fnr* coding and promoter sequences were introduced into the Δ*fnr* mutant to generate the complemented strain C-Δ*fnr* (Appendix S2, Figure S2a – c). String test results showed that both WT and C-Δ*fnr* strains produced a viscous string exceeding 5 mm in length when stretched from a colony (Figure 1(a)), while the Δ*fnr* mutant failed to exhibit this phenotype. Following centrifugation, the supernatants of the WT and C-Δ*fnr* strains remained turbid due to their mucoid nature, whereas the Δ*fnr* mutant formed a clear supernatant as bacterial cells settled at the bottom of the tube (Figure 1(b)). Quantitative analysis of the supernatant following centrifugation revealed that the Δ*fnr* mutant strain exhibited decreased supernatant turbidity, with its OD_600_ values being significantly lower compared to those of the WT and C-Δ*fnr* strains (*p* < 0.0001) (Figure 1(c)).

### Deletion of fnr attenuates resistance to phagocytosis and serum killing

**Figure 2.**
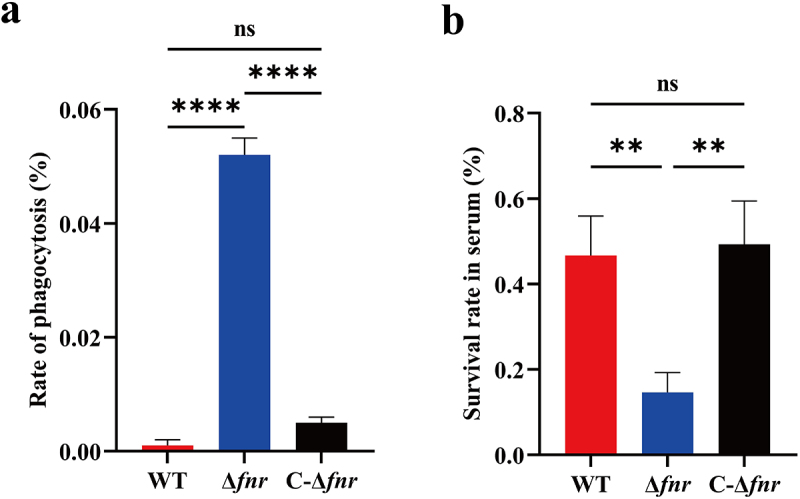
Deletion of *fnr* attenuates resistance to phagocytosis and serum killing. (a) In the phagocytosis assay, the WT and C-Δ*fnr* strains exhibited significantly greater resistance than the Δ*fnr* mutant. (b) In the serum killing assay, the WT and C-Δ*fnr* strains demonstrated markedly higher survival rates than the Δ*fnr* mutant. **p < 0.01, ****p < 0.0001, ns: not significant.

As the HMV phenotype confers protection against phagocytosis and serum killing in *K. pneumoniae*, we next evaluated whether FNR modulates these defense mechanisms. Phagocytosis assays revealed that the Δ*fnr* mutant was phagocytosed at significantly higher rates than the WT and C-Δ*fnr* (*p* < 0.0001) (Figure 2(a)). In parallel, serum killing assays demonstrated that the Δ*fnr* mutant had markedly reduced survival in healthy human serum compared with the WT and C-Δ*fnr* (*p* < 0.01) (Figure 2(b)). Together, these findings indicate that FNR enhances resistance to phagocytosis and serum killing in hvKP.

### Deletion of fnr inhibits capsule polysaccharide synthesis

**Figure 3.**
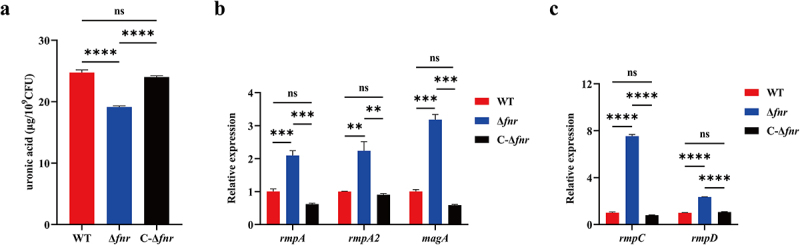
Deletion of *fnr* inhibits capsule polysaccharide synthesis. (a) Quantification of extracellular uronic acid demonstrated a significant reduction in capsular polysaccharide biosynthesis in the Δ*fnr* mutant compared to both the WT and C-Δ*fnr* strains. (b, c) mRNA expression levels of *rmpA*, *rmpA2*, *magA*, *rmpC*, and *rmpD* were upregulated in the Δ*fnr* mutant. **p < 0.01, ***p < 0.001, ****p < 0.0001, ns: not significant.

To assess the role of FNR in capsule biosynthesis, we quantified extracellular uronic acid and evaluated the expression of capsule- and HMV-associated genes. The WT and C-Δ*fnr* strains produced significantly more uronic acid than the Δ*fnr* mutant (*p* < 0.0001) (Figure 3(a)). Conversely, mRNA levels of *rmpA*, *rmpA2*, *magA*, *rmpC*, and *rmpD* were significantly elevated in the Δ*fnr* mutant compared to the WT and C-Δ*fnr* strains (*p* < 0.01) (Figure 3(b,c)). These findings suggest that while FNR promotes capsule production, it may have a negative regulatory effect on the transcription of key capsule or HMV-related genes.

### Deletion of fnr enhances fimbriae production, biofilm formation, and adhesion

**Figure 4.**
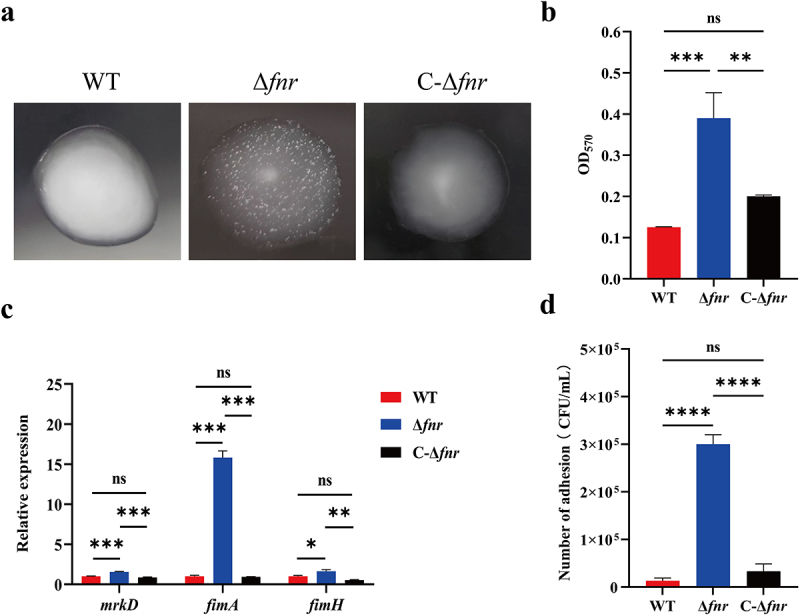
Deletion of *fnr* enhances fimbriae production, biofilm formation, and adhesion. (a) In the yeast agglutination assay, the Δ*fnr* mutant produced significantly more fimbriae than the WT and C-Δ*fnr* strains. (b) The mRNA expression levels of fimbriae-associated genes (*fimA*, *fimH*, and *mrkD*) were significantly greater in the Δ*fnr* mutant than in the WT and C-Δ*fnr* strains. (c) The Δ*fnr* mutant formed significantly more biofilm than the WT and C-Δ*fnr* strains, as measured by absorbance. (d) The Δ*fnr* mutant significantly outperformed the WT and C-Δ*fnr* strains in the cell adhesion assay. *p < 0.05, **p < 0.01, ***p < 0.001, ****p < 0.0001, ns: not significant.

Capsular components can modulate fimbriae-mediated adhesion and biofilm formation in *K. pneumoniae* [23]. Given that FNR positively regulates the HMV phenotype, we investigated whether FNR also influences fimbriae production, biofilm formation, and cellular adhesion. In the yeast agglutination assay, the Δ*fnr* mutant strain expressing strong type I fimbriae rapidly bound to mannose residues on *Saccharomyces cerevisiae* cells, resulting in visible particle aggregation. The Δ*fnr* mutant exhibited marked granule aggregation, whereas the WT and C-Δ*fnr* strains did not (Figure 4(a)). qRT-PCR analysis revealed that expression levels of the type I fimbriae genes *fimA* and *fimH* were significantly upregulated in the Δ*fnr* mutant compared to WT and C-Δ*fnr* strains (*p* < 0.05). In addition, the type III fimbriae gene *mrkD* was significantly overexpressed in the Δ*fnr* strain (*p* < 0.001) (Figure 4(c)). Biofilm-forming ability, assessed via crystal violet staining, was significantly higher in the Δ*fnr* mutant than in the WT and C-Δ*fnr* strains (*p* < 0.01) (Figure 4(b)). Furthermore, in A549 cell adhesion assays, the Δ*fnr* mutant adhered in significantly greater numbers than the WT and C-Δ*fnr* strains (*p* < 0.0001) (Figure 4(d)). Collectively, these findings suggest that FNR negatively regulates fimbriae production, biofilm formation, and bacterial adhesion in hvKP.

### Deletion of fnr attenuates the toxic effects of hvKP on *Galleria mellonella* larvae

**Figure 5.**
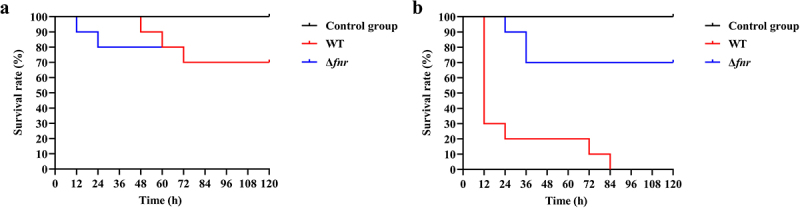
Deletion of *fnr* attenuates the toxic effects of hvKP on *Galleria mellonella* larvae. (a) Survival curves of *Galleria mellonella* larvae injected with 10^3^ CFU of hvKP strains. (b) Survival curves of *Galleria mellonella* larvae injected with 10^6^ CFU of hvKP strains.

To assess the impact of *fnr* deletion on overall virulence, we used *Galleria mellonella* larvae as an in vivo infection model. Larvae were injected with either a low (10^3^ CFU) or high (10^6^ CFU) bacterial dose of WT or Δ*fnr* strains, and survival was monitored over 120 hours. In the low-dose infection model (Figure 5(a)), survival remained comparable between groups, with 100% survival in the WT group and 90% in the Δ*fnr* group at 12 hours, and 80% in both groups at 60 hours. However, in the high-dose model (Figure 5(b)), survival differences were pronounced. At 12 hours, 30% of larvae survived in the WT group versus 100% in the Δ*fnr* group. By 24 hours, WT survival dropped to 20%, while Δ*fnr* survival remained at 90%. At 84 hours, all of the WT-infected larvae had died, whereas 70% of the Δ*fnr*-infected larvae survived. A log-rank test confirmed the statistical significance of these differences (*p* < 0.001). These data indicate that deletion of *fnr* significantly attenuates hvKP virulence in *G. mellonella* larvae.

### Deletion of fnr reduces blood invasiveness and antiphagocytic ability of hvKP in vivo

**Figure 6.**
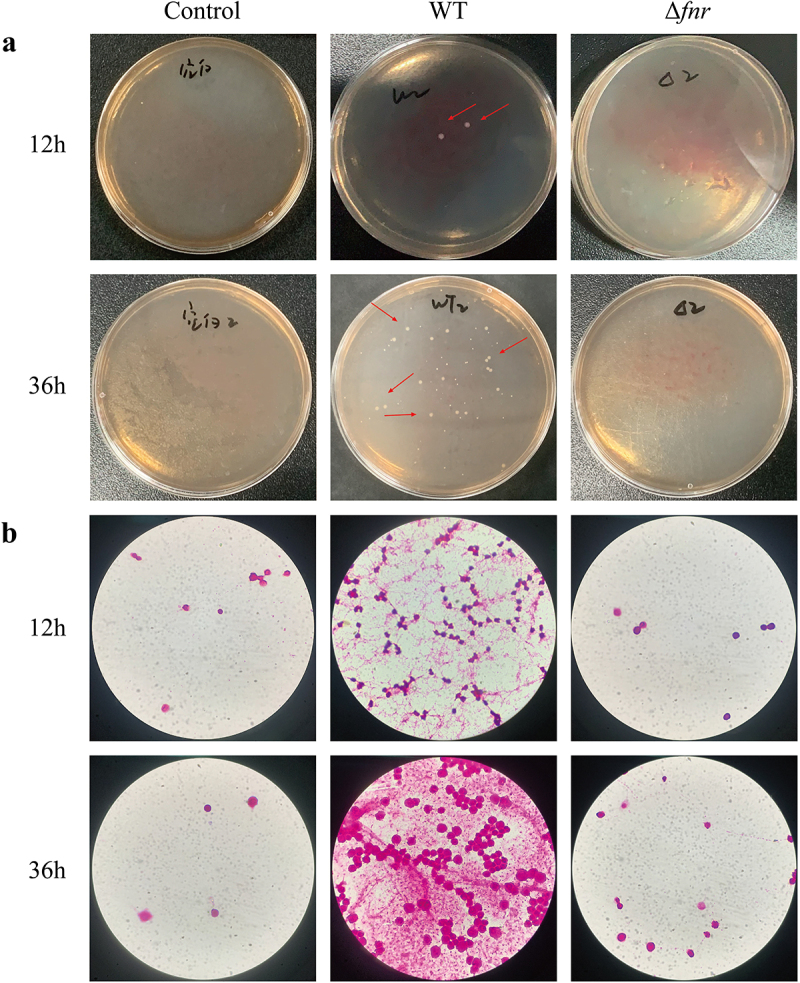
Deletion of *fnr* reduces the blood invasiveness and antiphagocytosis ability of hvKP in vivo. (a) Comparison of the bloodstream invasive capacity of the WT and Δ*fnr* strains. The WT group exhibited a marked increase in bacterial burden, rising from 12 ± 8 CFU/mL at 12 hours to 406 ± 40 CFU/mL at 36 hours (*p* < 0.0001). The Δ*fnr* mutant showed no detectable growth during the same period. Red arrows indicate representative colonies. Bacteria on the plates were biochemically identified as *K. pneumoniae*. (b) Microscopic examination of mouse intraperitoneal lavage fluid at 12 and 36 hours post-infection. Gram staining, magnification 1000 × .

To further characterize the in vivo virulence of the Δ*fnr* mutant, we examined bloodstream invasion and antiphagocytic capacity in a mouse model. Following intraperitoneal injection, ocular blood samples were collected at 12 and 36 hours post-infection. At 12 hours, bacterial colonies were observed in samples from the WT group, while none were detected in the Δ*fnr* group. By 36 hours, colony counts in the WT group increased significantly, reaching 406 ± 40 CFU/mL, while the Δ*fnr* group remained culture-negative (*p* < 0.0001) (Figure 6(a)). Concurrently, intraperitoneal lavage fluid was examined microscopically to assess phagocyte presence. At 12 hours, although *K. pneumoniae* was not visible in the WT group, the number of phagocytes was significantly higher than in the Δ*fnr* group, where only sparse phagocytes were noted. At 36 hours, the phagocyte count in the WT group increased further, and extracellular bacteria were visible. In contrast, there was minimal change in the phagocyte count in the Δ*fnr* group, and no bacteria were detected (Figure 6(b)). These results indicate that *fnr* deletion reduces both the bloodstream invasiveness and antiphagocytic capacity of hvKP in vivo.

## Discussion

The findings presented herein demonstrate the impact of *fnr* deletion on the hvKP strain NTUH-K2044 under both in vitro and in vivo conditions. These results provide robust evidence for the role of FNR in modulating key virulence-associated traits in hvKP and establish a foundation for future investigation into its underlying mechanisms. In the following discussion, we explore the regulatory functions of FNR, evaluate their implications for hvKP pathogenicity, and contextualize our findings within the broader literature.

FNR is a global transcriptional regulator that responds to anaerobic conditions and has been shown to control virulence in several bacterial species, including *Escherichia coli* [25–27], *Salmonella typhimurium* [19,28], and *Shigella* [29]. In this study, we found that FNR positively regulates the HMV phenotype in the hvKP strain NTUH-K2044. This regulation enhances bacterial resistance to phagocytosis and serum killing, while simultaneously suppressing fimbriae production, biofilm formation, and epithelial adhesion – traits associated with early colonization – ultimately contributing to increased virulence.

The HMV is a critical phenotypic feature of hvKP, playing a central role in immune evasion and the pathogenesis of invasive infections. In hvKP strains, *rmpA* is thought to activate the transcription of genes involved in capsular polysaccharide synthesis or HMW production, thereby promoting capsule formation or resulting in a mucoid phenotype [10,11,30,31]. In our study, the deletion of *fnr* led to the loss of the HMV phenotype and a significant reduction in extracellular polysaccharide (uronic acid) content, thus suggesting that FNR plays a crucial positive regulatory role in these traits in hvKP strains. However, when *fnr* was absent, the transcript levels of genes crucial for the HMV phenotype, including *rmpA*, *rmpA2*, *magA*, *rmpC*, and *rmpD*, were all increased. This seemingly contradictory outcome prompted us to investigate the underlying reasons. By analyzing the transcriptomes of WT and Δ*fnr* strains cultured under anaerobic conditions, we observed that, in addition to the genes mentioned above, the transcript levels of some genes in the *cps* cluster, as well as several genes related to lipopolysaccharide synthesis, were upregulated (data not shown). The inconsistency between the HMV phenotype and the expression of these polysaccharide synthesis-related genes led us to hypothesize that complex regulatory processes or feedback mechanisms may be involved. Further analysis of the transcriptome data revealed that the expression levels of genes associated with the phosphotransferase system (PTS) were generally downregulated in the *fnr*-deficient state, with several genes exhibiting particularly significant reductions. The PTS is a sugar transport system widely found in bacteria that is capable of actively transporting sugars into the cytoplasm [32]. We hypothesize that FNR, as a transcription factor, may directly or indirectly activate the transcription of PTS-related genes to promote sugar uptake and utilization, which is crucial for the development of the HMV phenotype in hvKP strains. When *fnr* is absent, cellular sugar transport is impaired, potentially triggering a compensatory mechanism. This mechanism may attempt to maintain the original phenotype and function by increasing the expression of polysaccharide-related genes. Future studies will rigorously examine the validity of this hypothesis.

The HMV phenotype is essential for the high pathogenicity of hvKP strains and is closely linked to invasive disease development [33]. A series of studies were conducted to confirm that the regulatory effect of FNR on the HMV phenotype of hvkP strains influences strain virulence. We initially carried out phagocytosis and serum killing assays, and the results corroborated our hypothesis that the loss of the HMV phenotype following *fnr* deletion diminishes hvKP’s resistance to phagocytosis and serum killing. This indicates that FNR positively modulates the evasion of two critical innate immune defenses. Our results diverge from those reported by Lin et al. [34], who demonstrated that in the *K. pneumoniae* strain CG43S3, a high producer of K2 serotype CPS [35], FNR negatively regulates antiphagocytic and antiserum activities by inhibiting CPS synthesis and the transcription of *rmpA* and *rmpA2*. Our study utilizing the hvKP strain NTUH-K2044, which produces K1 serotype CPS and exhibits a pronounced HMV phenotype, revealed that FNR positively modulates the HMV phenotype and capsule polysaccharide content. These opposing effects of FNR on bacterial virulence factors between the two strains underscore the strain-specific regulatory role of FNR in *K. pneumoniae*. The capsule and the extracellular mucus layer are important protective barriers for bacteria, and changes in either of them can greatly affect the resistance of bacteria to phagocytosis and serum killing. This divergence in FNR’s regulatory effects highlights the complexity of its role in bacterial pathogenesis and underscores the necessity for further research to elucidate the strain-specific regulatory mechanisms of FNR in different *K. pneumoniae* strains.

Bacterial colonization is a prerequisite for infection, prompting us to investigate the impact of FNR on the colonization capacity of the hvKP strain. Fimbriae are critical surface structures that mediate colonization and pathogenesis in *K. pneumoniae*, with type 1 (*fim*) and type 3 (*mrk*) fimbriae facilitating attachment to biotic and abiotic surfaces, thereby promoting biofilm formation and invasion [36]. However, strain-specific differences in colonization strategies exist; for example, the clinically relevant ST512 strain demonstrates poor biofilm formation on abiotic surfaces but exhibits strong adherence to eukaryotic cells [37]. These observations underscore the diverse mechanisms by which different strains adapt to host environments. Biofilm formation in *K. pneumoniae* is a multifactorial process influenced by environmental and genetic factors [38,39]. In our study, deletion of *fnr* increased fimbriae production, upregulated genes associated with both type I and type III fimbriae, enhanced biofilm formation, and improved cell adhesion. This is likely attributable to the loss of the HMV phenotype, which may otherwise mask fimbriae on the bacterial surface, thereby inhibiting adhesion [12,40]. The inverse relationship between extracellular capsule production and colonization-associated traits such as adhesion and biofilm formation has been well documented [40–42]. This reciprocal regulation enables the bacterium to fine-tune virulence factor expression across different stages of infection, maximizing adaptability and pathogenic potential. Supporting this notion, Cortes et al. [43] reported that unencapsulated *K. pneumoniae* mutants exhibit enhanced adhesion but reduced virulence in a murine pneumonia model. The positive regulation of the HMV phenotype in hvKP strains by FNR and the negative regulation of colonization and adhesion are not mutually exclusive. This regulatory mechanism may provide the bacterium with a highly adaptive mode of survival and infection, enabling it to maintain virulence and viability in diverse host environments.

Highly encapsulated *K. pneumoniae* strains are more virulent and capable of causing systemic infections. In the animal infection model, we observed that WT hvKP displayed dose-dependent virulence, whereas this effect was abolished in the Δ*fnr* mutant, highlighting the role of FNR in sustaining hypervirulence. Invasive syndromes caused by *K. pneumoniae* often involve bacteraemia, underscoring the importance of bloodstream invasion in bacterial transmission [44]. Our comparison of the blood invasion capabilities of the WT and Δ*fnr* mutant strains revealed that the WT strain invaded the bloodstream more rapidly and with a higher bacterial burden than the Δ*fnr* mutant. This enhanced invasiveness is likely attributable to the FNR-mediated upregulation of the HMV phenotype, which promotes immune evasion and enhances bacterial survival and proliferation within the host. Consistent with this, analysis of peritoneal lavage fluid revealed that WT infection led to significant phagocyte recruitment and the presence of extracellular bacteria over time, whereas the Δ*fnr* group showed minimal immune activation and no detectable bacteria. These findings illustrate the dynamic interaction between hvKP and host immunity. Despite a robust host immune response, the WT strain retained the ability to evade phagocytosis, underscoring the importance of FNR in modulating immune-evasive capabilities.

In conclusion, under anaerobic conditions, FNR positively regulates the HMV phenotype in hvKP, thereby enhancing bacterial resistance to immune defenses while concurrently diminishing bacterial colonization and adhesion. Furthermore, FNR significantly contributes to systemic virulence in animal models. This study provides evidence for the role of FNR in modulating bacterial pathogenicity and contributes to understanding the mechanisms underlying bacterial adaptation and survival within the host.

## Supplementary Material

Appendix S1 Successful generation of fnr gene deletion mutant.docx

Figure S2b.jpg

Table S1 Bacteria and plasmids used in the study.docx

Appendix S2 Successful establishment of the complemented strain.docx

Figure S2a.jpg

Figure S2c.jpg

Figure S1b.jpg

Figure S1e.jpg

Figure S1d.jpg

Figure S1a.jpg

Figure S1c.jpg

Table S2 Primers used in this study.docx

## Data Availability

The data that support the findings of this study are openly available in Zenodo at http://doi.org/10.5281/zenodo.15022844 , reference number [45].
