# Supplementary material for: Fumarate and nitrate reduction regulator (FNR) modulates hypermucoviscosity and virulence in hypervirulent Klebsiella pneumoniae through anaerobic adaptation
Source: Virulence. 2025 Jul 28;16(1):2536186. doi: 10.1080/21505594.2025.2536186 (PMC12309544; doi:10.1080/21505594.2025.2536186)
Supplement: Appendix S1 Successful generation of fnr gene deletion mutant.docx [file KVIR_A_2536186_SM6179.docx]

**Appendix S1.** **Successful generation of *fnr* gene deletion mutant.**

The upstream and downstream homology arms of *fnr* were amplified by PCR using the genomic DNA of strain NTUH K2044 as a template. As illustrated in **Figure S1a**, lane 1 represents the upstream homology arm of 951 bp amplified using the primer pair FNR-H73-F/FNR-up-R, while lane 2 shows the downstream homology arm of 894 bp amplified with the primer pair FNR-down-F/FNR-H73-R. These results are consistent with the expected fragment sizes. The successful amplification of both homology arms was confirmed, and their sizes matched the anticipated lengths. Subsequently, the upstream and downstream homology arms were fused via fusion PCR. As shown in **Figure S1b**, lane 1 displays the amplified fusion fragment FNR-UD-H73, which is 1845 bp in length, aligning with the expected size.


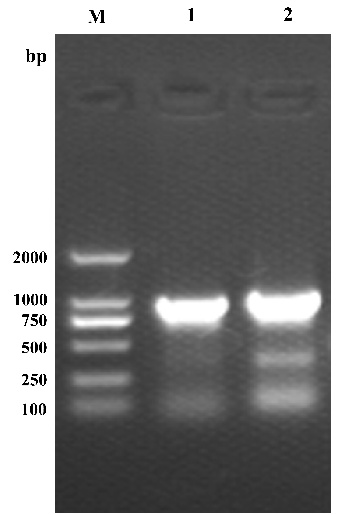


**Figure S1a. Electrophoretic pattern of the upstream and downstream homologous arms of *fnr*.** M: DL2000 Marker; Lane 1: homologous arm upstream of *fnr*; Lane 2: the downstream homologous arm of *fnr*.


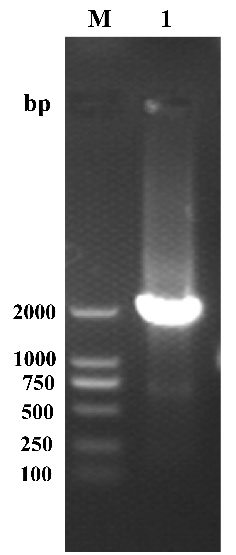


**Figure S1b. Electrophoresis of the upstream and downstream fusion fragment FNR-UD-H73.** M: DL2000 Marker; Lane 1: Fusion fragment FNR-UD-H73.

The ligation product, derived from the seamless clonal ligation of the FNR-UD-H73 homology arm fusion fragment with the *Sma* I-linearized suicide plasmid vector pH73, was verified through colony PCR using primer pairs FNR-JD-F/FNR-JD-R, as illustrated in **Figure S1c**. The expected amplicon size for a positive clone is 612 bp. All six randomly selected monoclonal clones yielded results consistent with this expectation.

~~
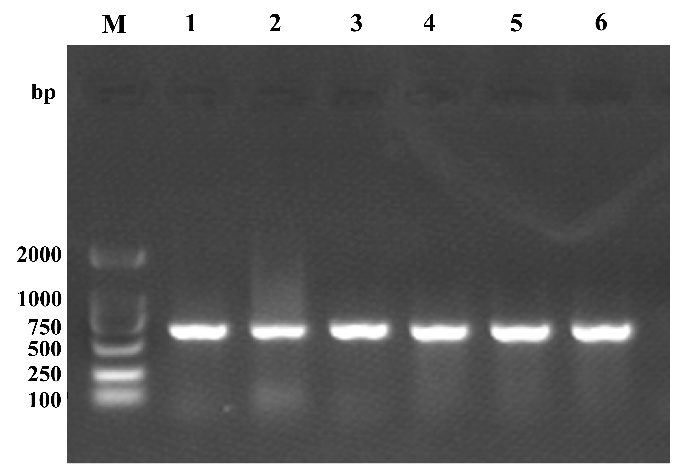
~~

**Figure S1c.** PCR identification of the connected product colonies. M: DL2000 Marker; Lane 1-6: Six randomly selected monoclonal lines were amplified using the primer pair FNR-JD-F and FNR-JD-R.

The recombinant plasmid FNR-pH73 S17 λ*pir* was successfully transferred into NTUH K2044 via conjugation. Subsequently, the *fnr* mutant strain was obtained through sucrose-induced double-crossover screening. As illustrated in **Figure S1d**, PCR amplification of the wild-type strain using the primer pair FNR-JD-F/FNR-JD-R yielded a 1365 bp fragment, whereas the mutant strain produced a 612 bp homology arm fragment, indicating the absence of a 753 bp *fnr* gene fragment. Amplification of the wild-type strain with the primer pair FNR-ter-F/FNR-ter-R resulted in the acquisition of a 753 bp fragment corresponding to the *fnr* gene, as shown in **Figure S1e**. In contrast, no amplification product was observed for the mutant strain.


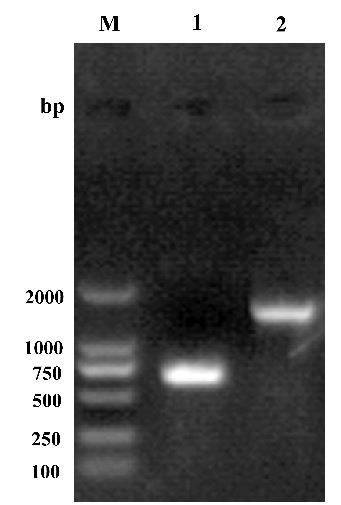


**Figure S1d. Electrophoretic images of mutant and wild strains amplified by FNR-JD-F/FNR-JD-R.** M: DL2000 Marker; Lane 1: *fnr* mutant; Lane2: wild type strain


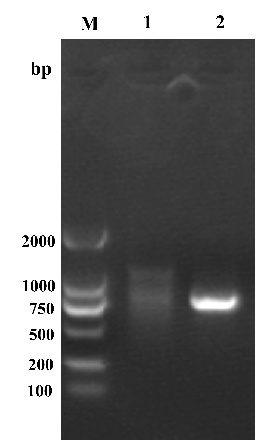


**Figure S1e. Electrophoresis of FNR-ter-F/FNR-ter-R amplified mutant and wild strain.** M: DL2000 Marker; Lane 1: *fnr* mutant; Lane2: wild type strain
