# Supplementary figures and images for: Fumarate and nitrate reduction regulator (FNR) modulates hypermucoviscosity and virulence in hypervirulent Klebsiella pneumoniae through anaerobic adaptation
Source: Virulence. 2025 Jul 28;16(1):2536186. doi: 10.1080/21505594.2025.2536186 (PMC12309544; doi:10.1080/21505594.2025.2536186)

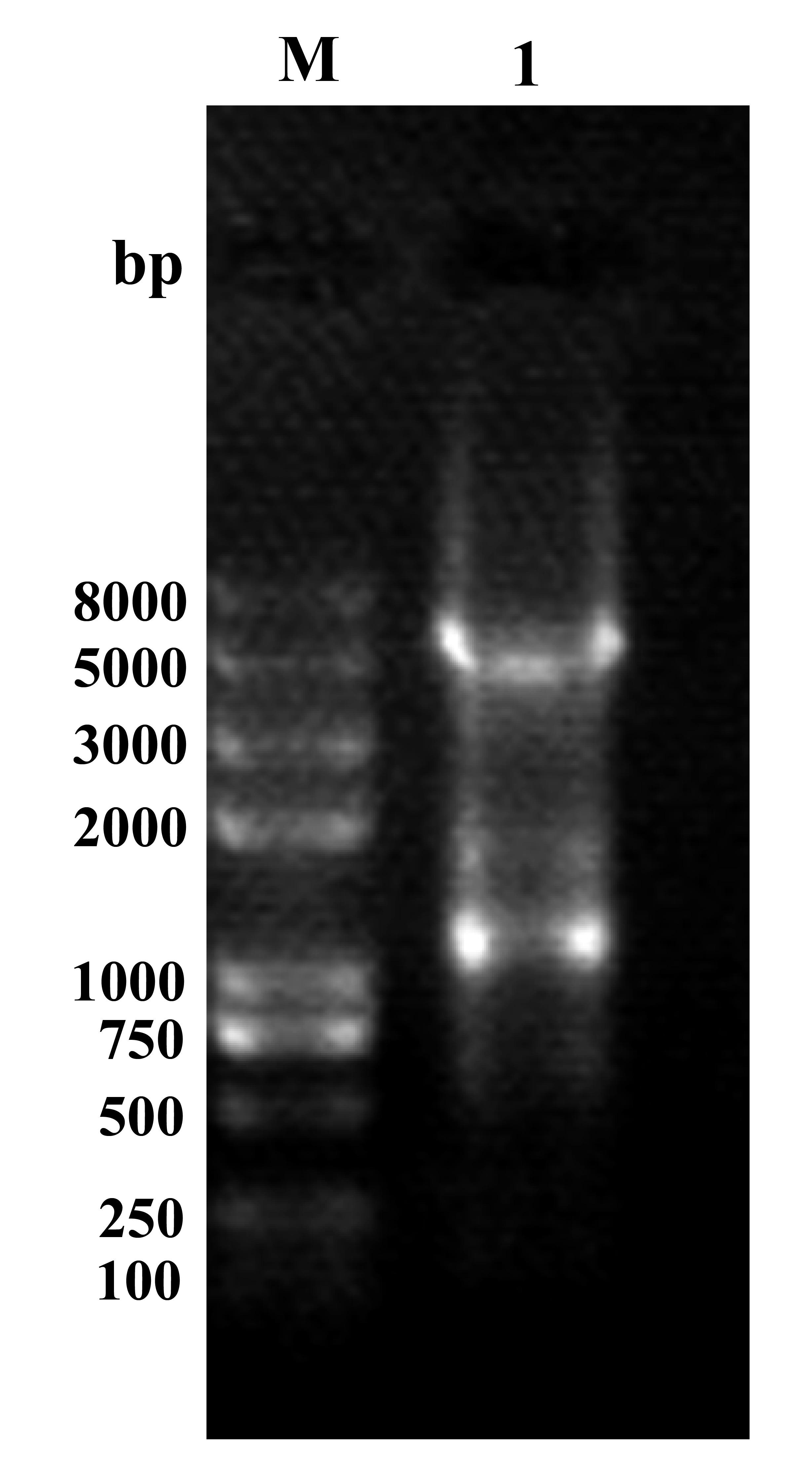

Supplement: Figure S2b.jpg [file KVIR_A_2536186_SM6178.jpg]

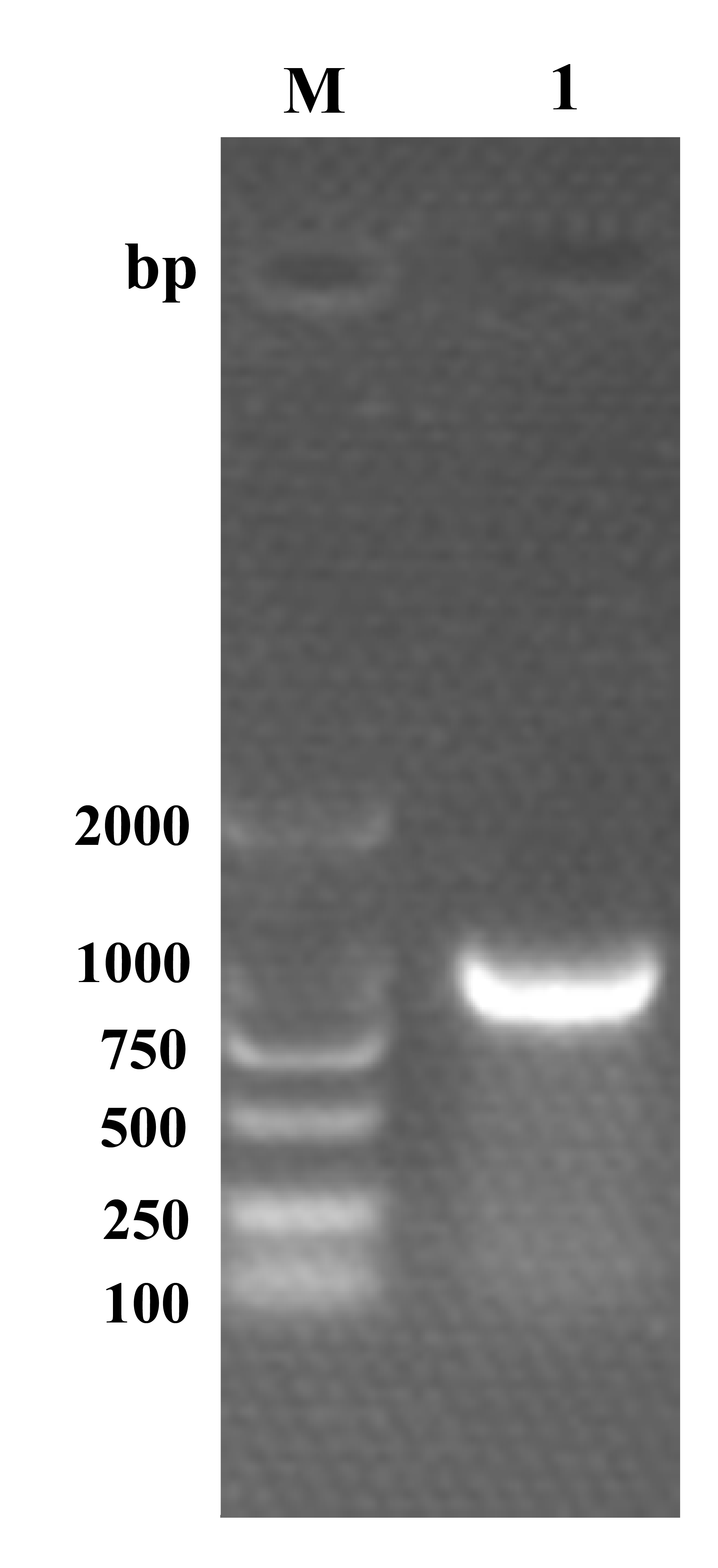

Supplement: Figure S2a.jpg [file KVIR_A_2536186_SM6175.jpg]

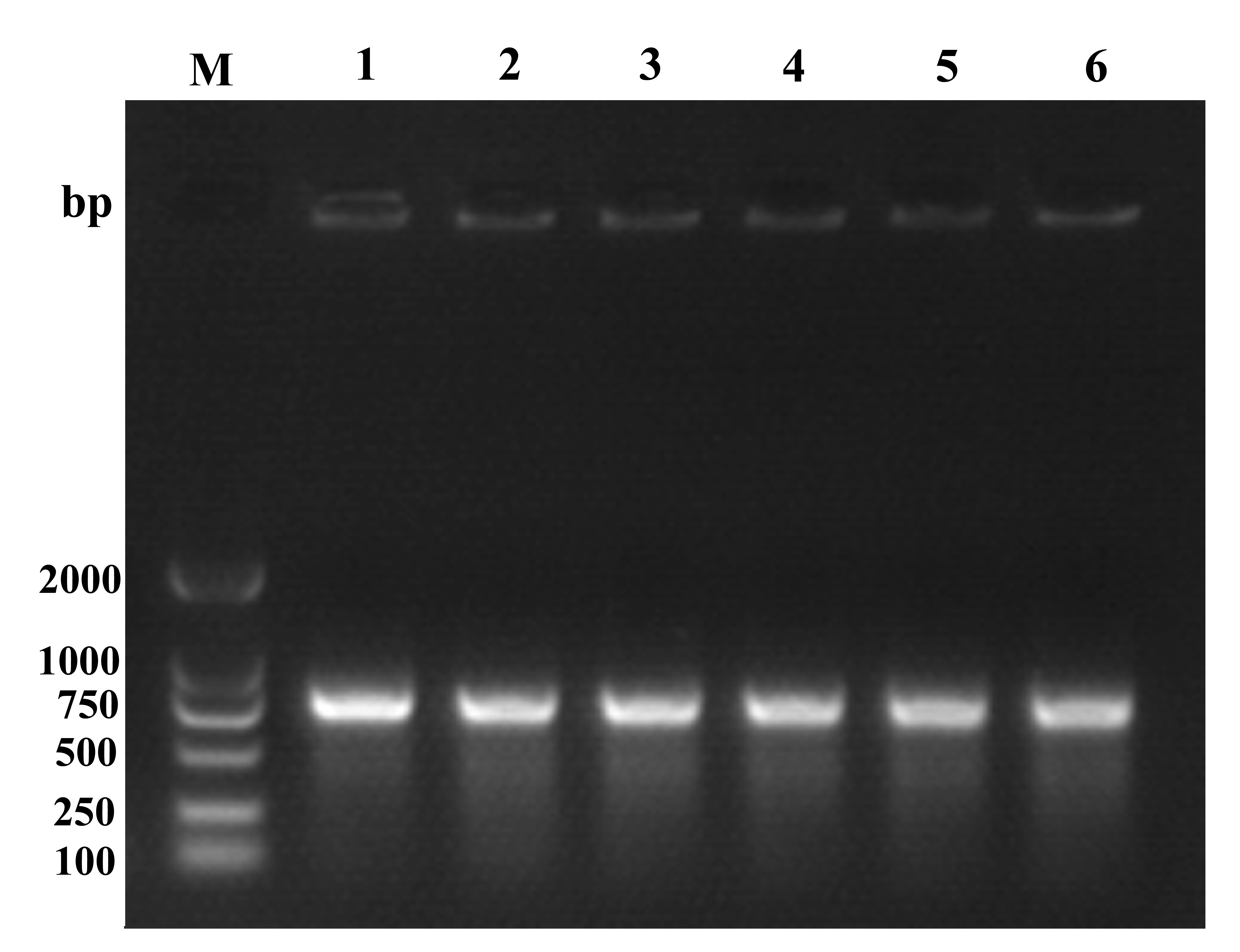

Supplement: Figure S2c.jpg [file KVIR_A_2536186_SM6174.jpg]

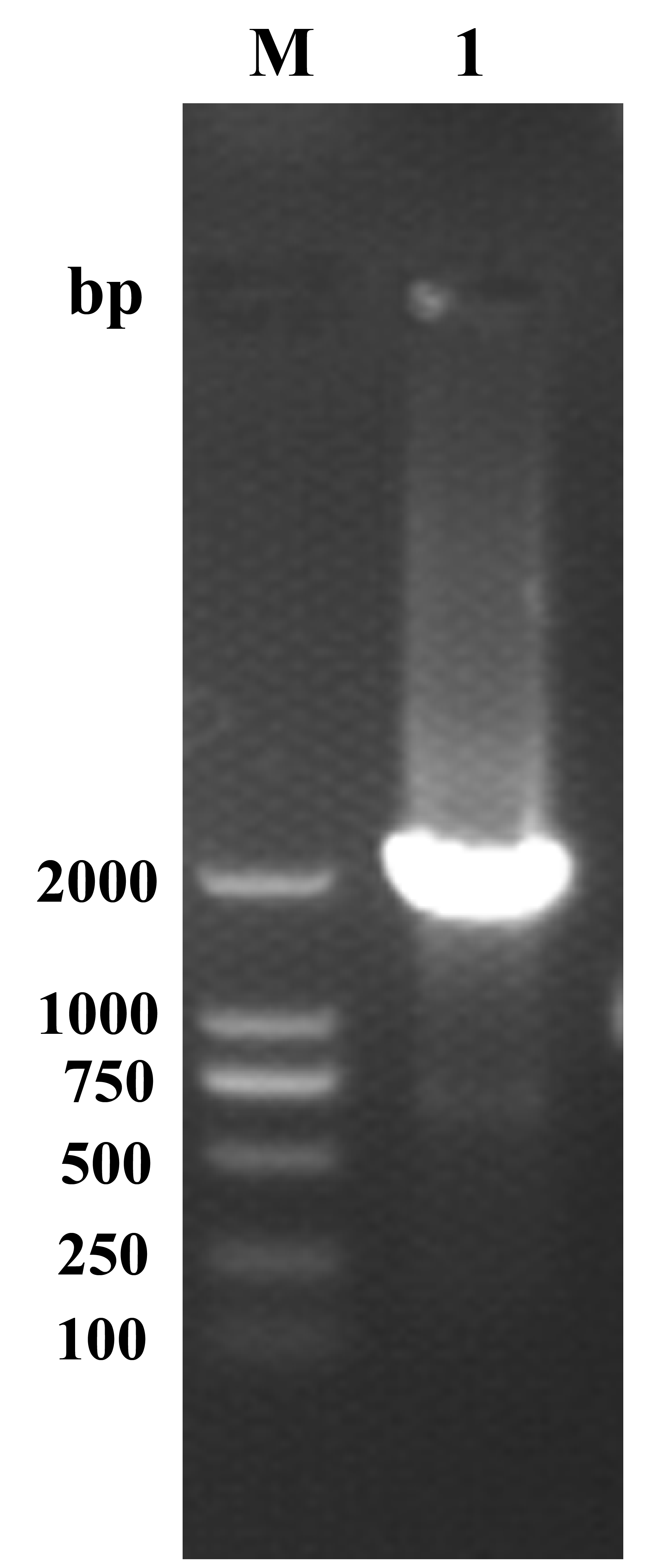

Supplement: Figure S1b.jpg [file KVIR_A_2536186_SM6173.jpg]

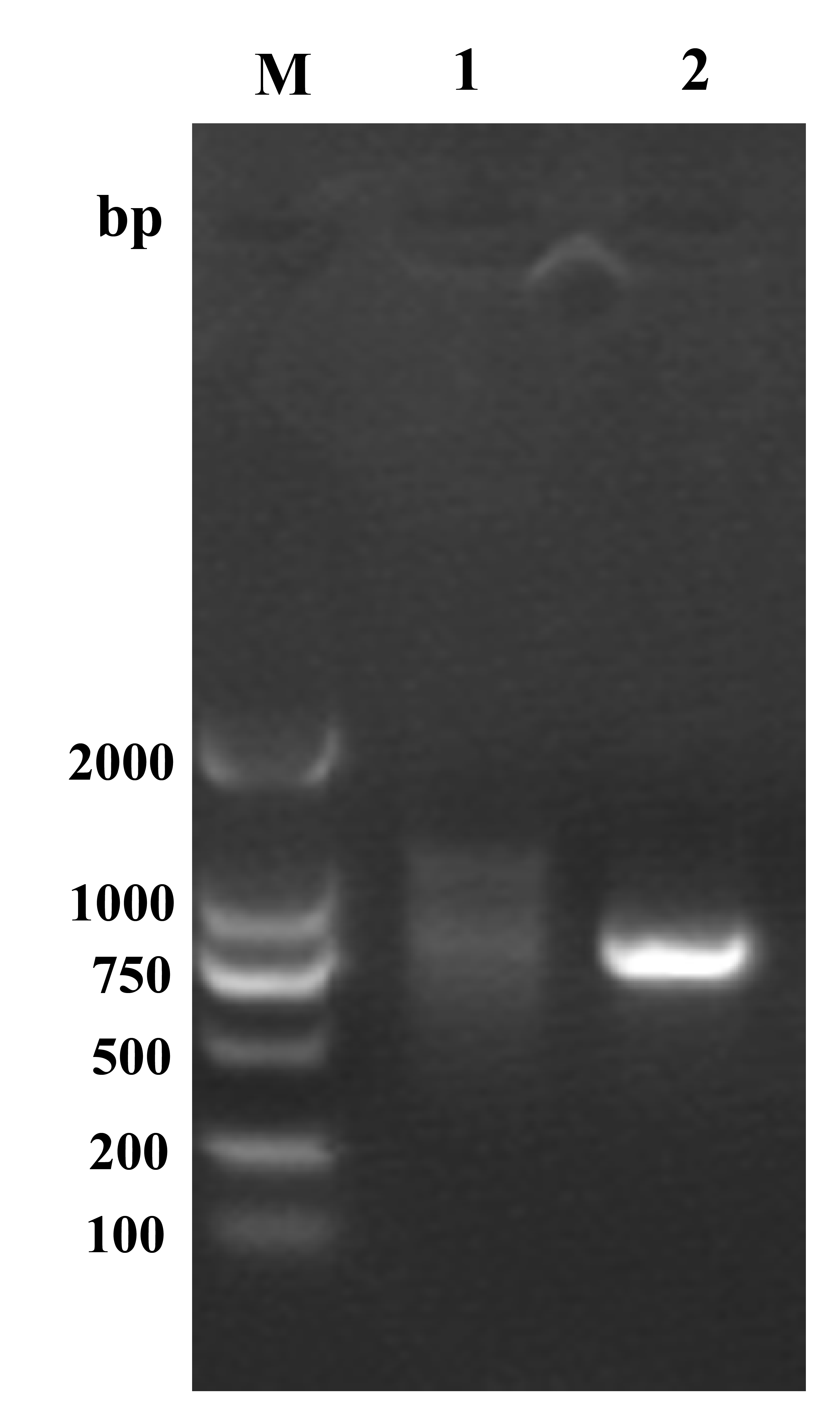

Supplement: Figure S1e.jpg [file KVIR_A_2536186_SM6172.jpg]

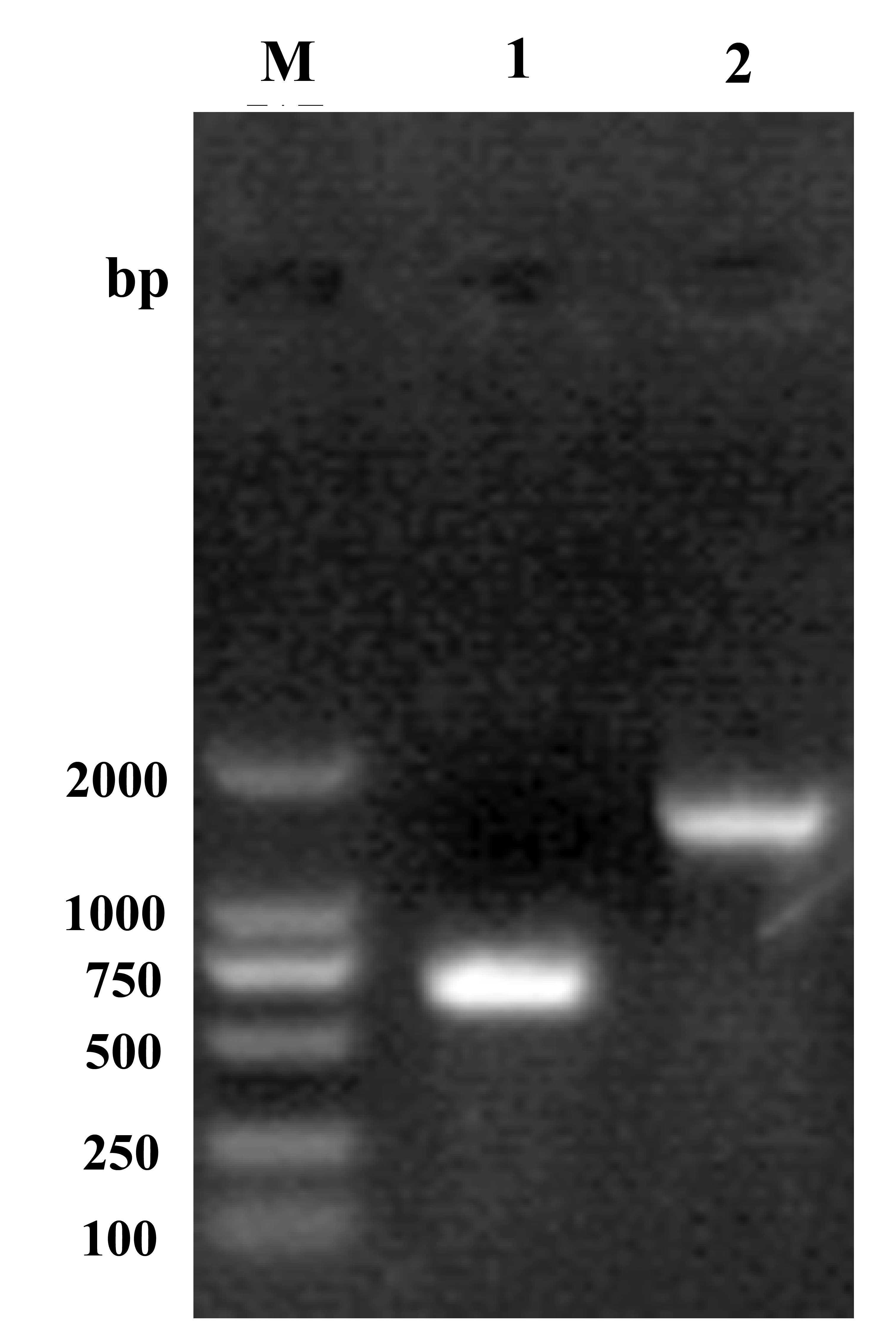

Supplement: Figure S1d.jpg [file KVIR_A_2536186_SM6171.jpg]

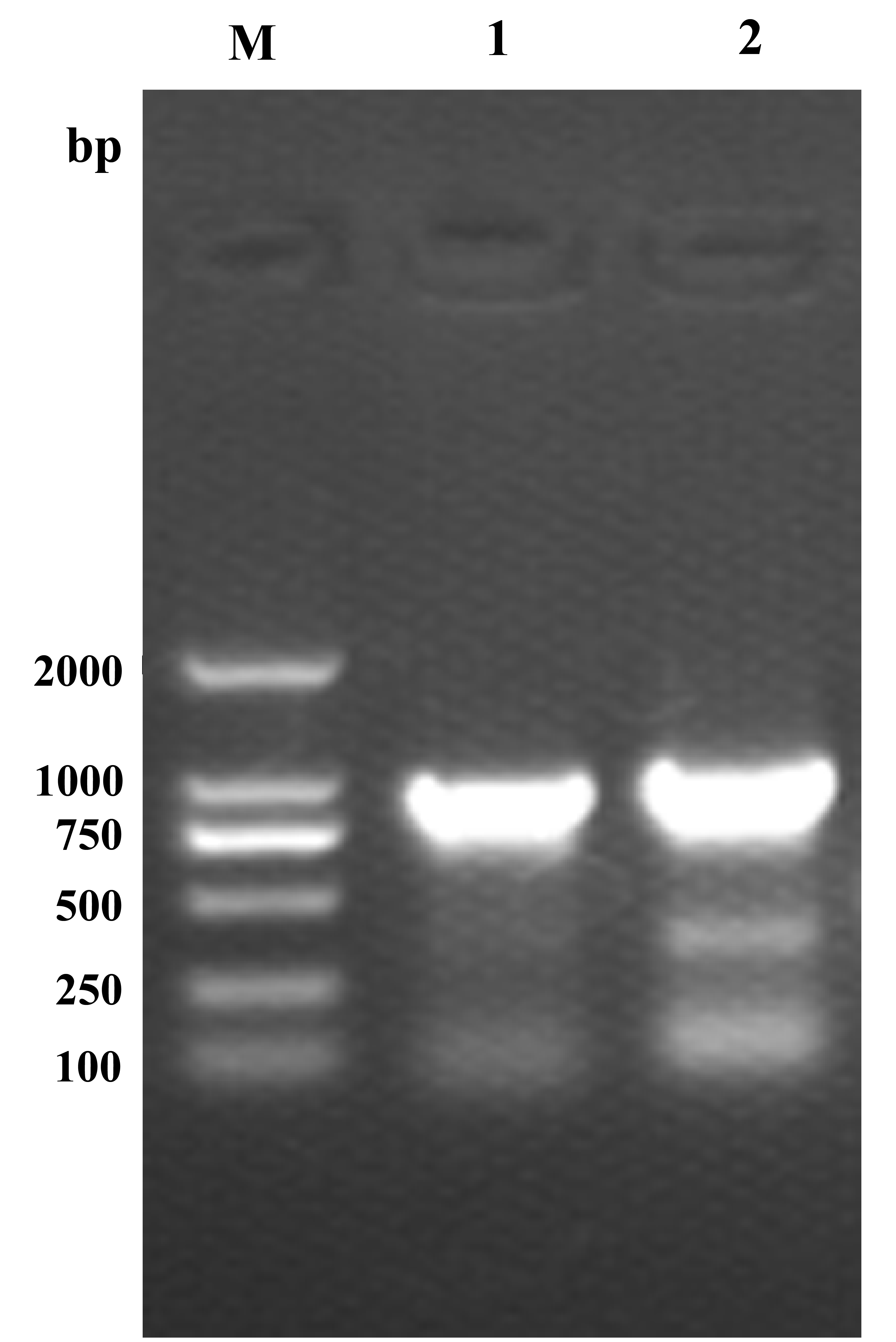

Supplement: Figure S1a.jpg [file KVIR_A_2536186_SM6170.jpg]

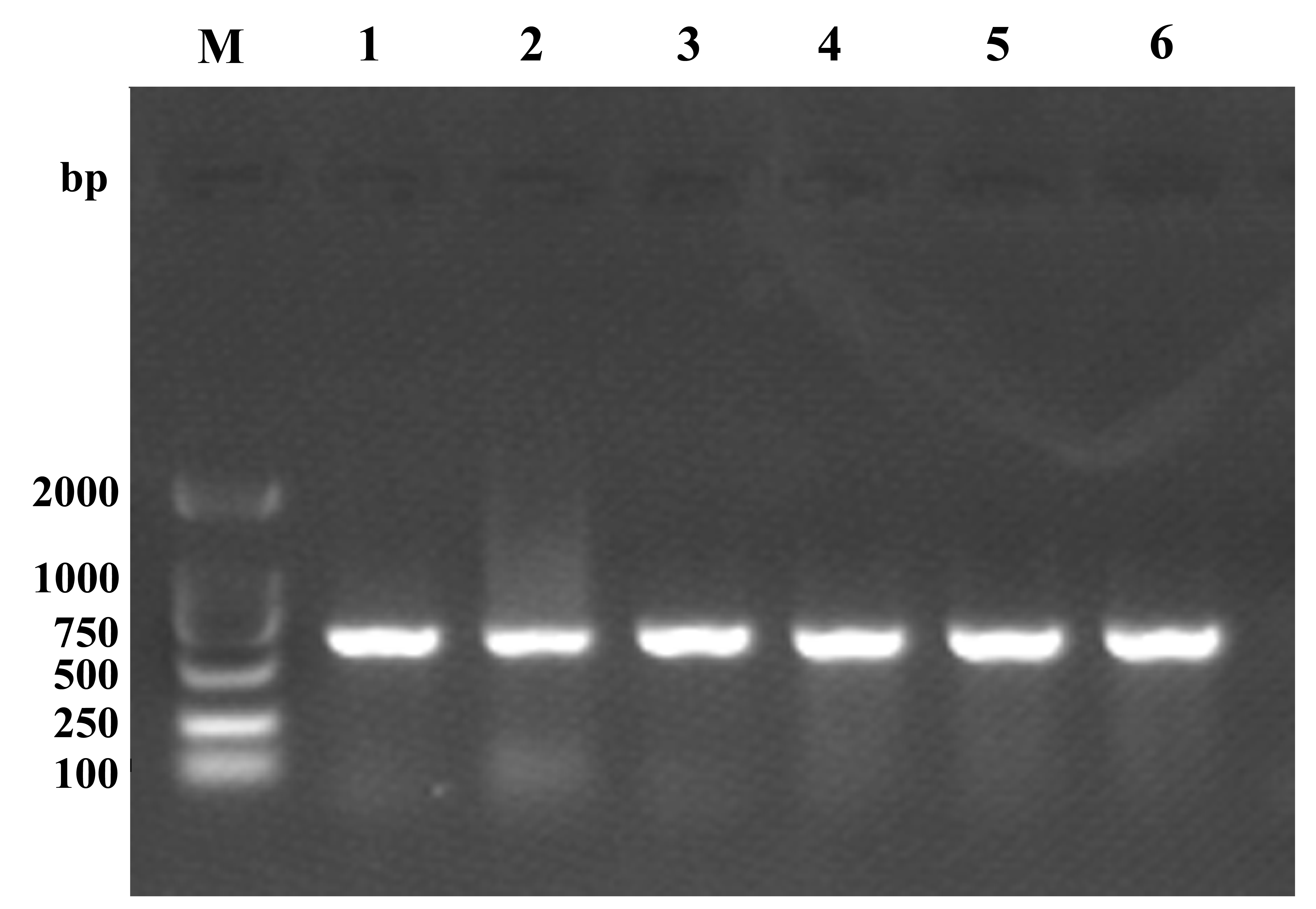

Supplement: Figure S1c.jpg [file KVIR_A_2536186_SM6169.jpg]
