# Supplementary material for: Fumarate and nitrate reduction regulator (FNR) modulates hypermucoviscosity and virulence in hypervirulent Klebsiella pneumoniae through anaerobic adaptation
Source: Virulence. 2025 Jul 28;16(1):2536186. doi: 10.1080/21505594.2025.2536186 (PMC12309544; doi:10.1080/21505594.2025.2536186)
Supplement: Table S1 Bacteria and plasmids used in the study.docx [file KVIR_A_2536186_SM6177.docx]

**Table S1** Bacteria and plasmids used in the study.

| **Strains or plasmids** | **Descriptions** | **Reference or source** |
| --- | --- | --- |
| ***K. pneumoniae*** |  |  |
| NTUH K2044 | K2044 Amp^r^ | Laboratory stock |
| Δ*fnr* | K2044 Δ*fnr* | This study |
| C-Δ*fnr* | K2044 C-Δ*fnr* | This study |
| ***E. coli*** |  |  |
| DH5α | deoR endA1 gyrA96 hsdR17 (rk-mk+)recA1 relA1 supE44 thi-1Δ(lacZYA-argF)U169 Φ80lacZ ΔM15F -λ- | Laboratory stock |
| S17-1 λ *pir* | *hsdR recA pro* RP4-2 [Tc:Mu; Km:Tn*7*] [λ*pir*] | Laboratory stock |
| **Plasmids** |  |  |
| pH73 | Cm^r^， Sma I restriction endonuclease single-enzyme linearized suicide vector， *sacB*. | Laboratory stock |
| pB95 | Cm^r^, low-copy-number cloning vector | Laboratory stock |
