# Supplementary material for: Fumarate and nitrate reduction regulator (FNR) modulates hypermucoviscosity and virulence in hypervirulent Klebsiella pneumoniae through anaerobic adaptation
Source: Virulence. 2025 Jul 28;16(1):2536186. doi: 10.1080/21505594.2025.2536186 (PMC12309544; doi:10.1080/21505594.2025.2536186)
Supplement: Appendix S2 Successful establishment of the complemented strain.docx [file KVIR_A_2536186_SM6176.docx]

**Appendix S2.** **Successful establishment of the complemented strain.**

The genomic DNA from NTUH K2044 was used as a template to successfully amplify the *fnr* gene and its promoter region using the FNR-B95-F/FNR-B95-R primer pair, as illustrated in **Figure S2a**. Additionally, the linearized pB95 plasmid was amplified using the B95-FNR-F/B95-FNR-R primers with the pB95 plasmid as a template, as demonstrated in **Figure S2b**. The recombinant plasmid pB95-FNR was constructed by ligating the linearized pB95 plasmid with the amplified *fnr* and promoter fragments via seamless cloning. Subsequently, this recombinant plasmid was introduced into *fnr* mutant strains through electroporation, resulting in the generation of *fnr*-complemented strains. For verification, the FNR-ter-F/FNR-ter-R primer pair was used, and as shown in **Figure S2c**, all six randomly selected monoclones yielded an expected amplicon size of 753 bp.


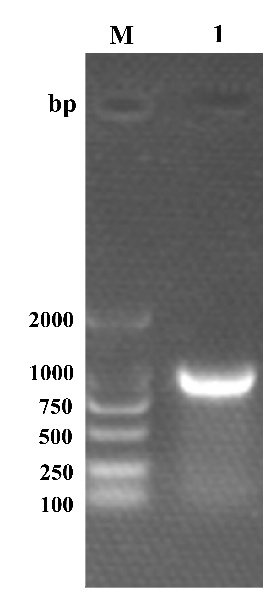


**Figure S2a. The electropherograms of *fnr* and promoter fragments.** M: DL2000 Marker; Lane 1: *fnr* and promoter fragments


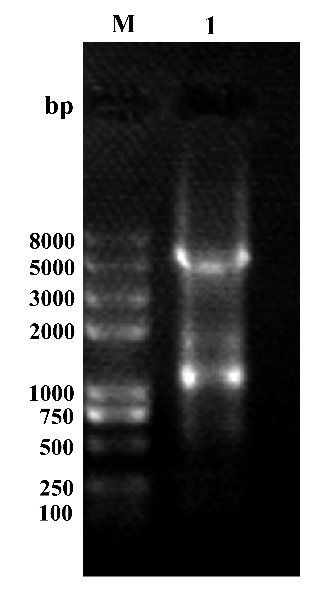


**Figure S2b. The electropherogram of reverse amplified pB95 plasmid.** M: DL8000 Marker; Lane 1: Linearized pB95 plasmid


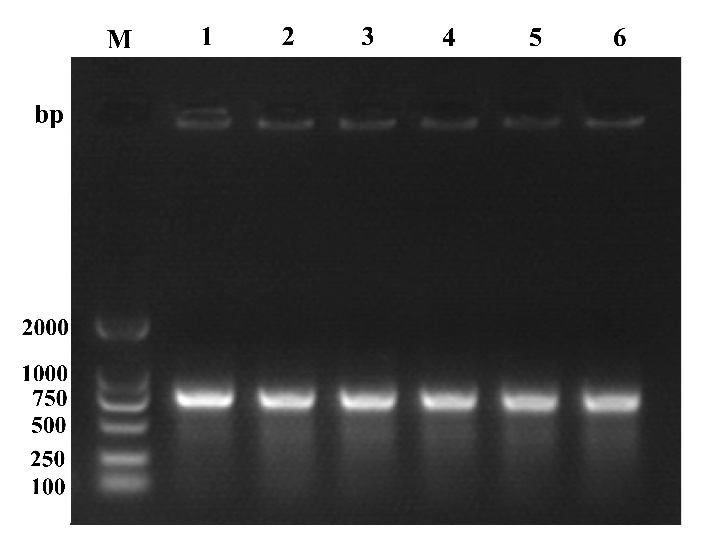


**Figure S2c. Electrophoretic identification of *fnr* complemented strain by PCR.** M: DL2000 Marker; Lane1-6:Six randomly selected clones were amplified with FNR-ter-F/FNR-ter-R as primers.
