# Supplementary material for: Fumarate and nitrate reduction regulator (FNR) modulates hypermucoviscosity and virulence in hypervirulent Klebsiella pneumoniae through anaerobic adaptation
Source: Virulence. 2025 Jul 28;16(1):2536186. doi: 10.1080/21505594.2025.2536186 (PMC12309544; doi:10.1080/21505594.2025.2536186)
Supplement: Table S2 Primers used in this study.docx [file KVIR_A_2536186_SM6168.docx]

**Table S2** Primers used in this study.

| **Primers** | **Sequence (5'-3')** | **Purpose** |
| --- | --- | --- |
| FNR-H73-F | AAGTGAACTGCATGAATTGACCGTTCCCAGCACCACGATCC | Amplification of the upstream homologous arm of *fnr* |
| FNR-up-R | TAAGGCCTGAGCAGACATTCTCTGCGCAATACCTGGTCAC |  |
| FNR-down-F | CCAGGTATTGCGCAGAGAATGTCTGCTCAGGCCTTAATTG | Amplification of the downstream homologous arm of *fnr* |
| FNR-H73-R | ATATCGAGCTCTCCCGGGCCTGTCCGGTAACCCGCCAGGAT |  |
| FNR-ter-F | TTAAGCGACGTTGCGCGCCTGCC | Identification of the *fnr* mutant strain |
| FNR-ter-R | ATGATCCCTGAGAAGCGAATTAT |  |
| FNR-JD-F | ATTCCAGGTAGAATTTCGCCTGC | Identification of the *fnr* mutant strain |
| FNR-JD-R | TGGCTTTTGCGTCATGAGGGCTA |  |
| FNR-B95-F | CGGTAGTCAATAAACCGGTGGCTATCTGTTGCTTTGACTG | Amplification of *fnr* and its promoter |
| FNR-B95-R | TTAATGGTGATGGTGATGGTGAGCGACGTTGCGCGCCTGC |  |
| B95-FNR-F | ACCATCACCATCACCATTAACAGCTTTTGTTCCCTTTAGT | Reverse amplification of pB95 plasmid vector |
| B95-FNR-R | TCAAAGCAACAGATAGCCACCGGTTTATTGACTACCGGAA |  |
| pB95-JD-F | GAAGCCGGCGGCACCTCGCTAAC | identification of *fnr* complementary strain |
| M13-JD-R | AGCGGATAACAATTTCACACAGG |  |
| **For qRT-PCR Primers** | **Sequence (5'-3')** | **Target** |
| *16S rRNA*-F | AGCCGACCTGAGAGGGTGA | *16S rRNA* |
| *16S rRNA*-R | TCTGGACCGTGTCTCAGTTCC |  |
| *mrkD*-F | CCACCAACTATTCCCTCGAA | *mrkD* |
| *mrkD*-R | ATGGAACCCACATCGACATT |  |
| *fimH*-F | GCCAACGTCTACGTTAACCTG | *fimH* |
| *fimH*-R | ATATTTCACGGTGCCTGAAAA |  |
| *fimA*-F | GTTCTGGCCCTGCAAAACTC | *fimA* |
| *fimA*-R | TGGAACGGGATGATGTTGGG |  |
| *rmpA2*-F | ACGTATGAAGGCTCGATGGAT | *rmpA2* |
| *rmpA2*-R | AACCATCCCATTTCCCTGAAT |  |
| *rmpA*-F | AGAGTATTGGTTGACAGCAGGA | *rmpA* |
| *rmpA*-R | ACGTCAAGCCACATCCATTG |  |
| *magA*-F | GAGCAATATGGCCAGTCCGA | *magA* |
| *magA*-R | TTCCCACTCCCTCTCCAAGT |  |
| *rmpC*-F | AGCTAAGTGGATGCCAAGGA | *rmpC* |
| *rmpC*-R | CCCTTTTAACTCACACACGGA |  |
| *rmpD*-F | AATGTTCTGTGCGAGCGGA | *rmpD* |
| *rmpD*-R | AGGAGGGGGTGAAAGCACTC |  |
